# Embedding Functional Brain Networks in Low Dimensional Spaces Using Manifold Learning Techniques

**DOI:** 10.3389/fninf.2021.740143

**Published:** 2021-12-24

**Authors:** Ramon Casanova, Robert G. Lyday, Mohsen Bahrami, Jonathan H. Burdette, Sean L. Simpson, Paul J. Laurienti

**Affiliations:** ^1^Department of Biostatistics and Data Science, Wake Forest School of Medicine, Winston-Salem, NC, United States; ^2^Laboratory for Complex Brain Networks, Wake Forest School of Medicine, Winston-Salem, NC, United States; ^3^Department of Radiology, Wake Forest School of Medicine, Winston-Salem, NC, United States

**Keywords:** brain networks, UMAP, t-SNE, manifold learning, machine learning

## Abstract

**Background:** fMRI data is inherently high-dimensional and difficult to visualize. A recent trend has been to find spaces of lower dimensionality where functional brain networks can be projected onto manifolds as individual data points, leading to new ways to analyze and interpret the data. Here, we investigate the potential of two powerful non-linear manifold learning techniques for functional brain networks representation: (1) T-stochastic neighbor embedding (t-SNE) and (2) Uniform Manifold Approximation Projection (UMAP) a recent breakthrough in manifold learning.

**Methods:** fMRI data from the Human Connectome Project (HCP) and an independent study of aging were used to generate functional brain networks. We used fMRI data collected during resting state data and during a working memory task. The relative performance of t-SNE and UMAP were investigated by projecting the networks from each study onto 2D manifolds. The levels of discrimination between different tasks and the preservation of the topology were evaluated using different metrics.

**Results:** Both methods effectively discriminated the resting state from the memory task in the embedding space. UMAP discriminated with a higher classification accuracy. However, t-SNE appeared to better preserve the topology of the high-dimensional space. When networks from the HCP and aging studies were combined, the resting state and memory networks in general aligned correctly.

**Discussion:** Our results suggest that UMAP, a more recent development in manifold learning, is an excellent tool to visualize functional brain networks. Despite dramatic differences in data collection and protocols, networks from different studies aligned correctly in the embedding space.

## Impact Statement

We investigate the potential of two powerful non-linear manifold learning techniques for functional brain network representation: t-SNE and UMAP. While t-SNE has received attention by the fMRI community for some time, UMAP is a much more recent development. Here, we investigate the potential of these two techniques when embedding fMRI brain network data from different studies into a common 2D space despite differences in acquisition protocols. These techniques are actively being used in the field and we expect our study will provide useful information to the neuroimaging community regarding their use.

## Introduction

For the past 30 years, the generation, analysis, representation, and, especially, interpretation of fMRI data has been challenging. With the advent of using fMRI to determine brain connectivity, this challenge has just magnified. fMRI data is inherently high-dimensional and difficult to visualize. As such, a recent trend in the neuroimaging community has been to find spaces of lower dimensionality where the fMRI data corresponding to multiple individuals can be projected onto manifolds as data points, thereby facilitating the identification of patterns within a given group of individuals and allowing new ways to analyze and interpret the data. Reducing the dimensionality of the data is critical for many applications as it allows avoiding redundancy, compact visualization and finding latent features in the data. Here, we investigate the potential of two powerful non-linear manifold learning techniques for functional brain networks representation. These two techniques are: (1) T-stochastic neighbor embedding (t-SNE) introduced by Van Maaten and Histon (2008) and (2) Uniform Manifold Approximation Projection (UMAP), a recent breakthrough in manifold learning developed by McInnes et al. (2018). We were specifically interested in their capabilities to represent functional brain networks from one group or study based on the learned low-dimensional mapping from a different dataset.

T-stochastic neighbor embedding has become popular in omics where it has been applied, for example, to single cell transcriptomics (Kobak and Berens, 2019), a field booming with developments in manifold learning. Recently, several groups have begun applying t-SNE to analyze neuroimaging data. Our group investigated the value of embedding brain fMRI dynamic networks in a low dimensional manifold using t-SNE (Bahrami et al., 2019). We were able to show that these low dimensional manifolds contain meaningful information, as they were able to successfully discriminate between cognitive tasks and study populations. Hu et al. (2020) used t-SNE to create an optimized framework that combines automatic spectral clustering with dimensionality reduction for fine-grained functional parcellation of resting-state fMRI (rs-fMRI) of the human brain. Saggar et al., used topological data analysis (Carlsson, 2009) combined with t-SNE to reveal the overall organization of whole-brain activity maps at a single-participant level without arbitrarily collapsing the data (Saggar et al., 2018). Using existing multitask fMRI datasets, their approach tracks both within- and between-task transitions at a fast time scale. They reported that individual differences in the revealed dynamical organization predicted task performance. Panta et al. (2016) have proposed t-SNE as a tool for visualization and quality control of structural and functional MRI as well. Tseng and Poppenk introduced a method based on independent component analysis and t-SNE to identify breaks between stable periods of brain network configuration or meta-state transitions at a single-TR timescale and using rs-fMRI data from single participants (Tseng and Poppenk, 2020). UMAP is a newer manifold learning technique for visualization, and this dimension reduction algorithm has been less applied to neuroimaging data to date. UMAP has been previously used by Gomez et al. (2020) to characterize temporally independent functional modes, which are functional brain networks identified based on their temporal independence.

The main goal of this work is to evaluate the potential of these manifold learning techniques to embed functional brain network data generated in different studies into a common 2D space. Another objective of this work is to gain some understanding about the differences between UMAP and t-SNE when used to visualize and interpret functional brain networks in 2D space. UMAP is an approach to deal with high-dimensional data that is based on topological principles. It is becoming increasingly popular in bioinformatics and machine learning communities since several studies (Becht et al., 2018; McInnes et al., 2018) have suggested it scales better to high dimensional problems (in terms of sample size and number of variables) and produces more stable results than the more widely used t-SNE, which has been the state of the art for high-dimensional data visualization for several years (Van Maaten and Histon, 2008; Kobak and Berens, 2019). In addition, t-SNE was not designed for dimension reduction but rather for visualization purposes, with it being unclear its value for dimension reduction beyond 3D. While both methods aim to preserve local structure present in the high-dimensional data, UMAP developers have claimed that UMAP better preserves global structure (Becht et al., 2018). However, it seems that there is no agreement about the superiority of UMAP over t-SNE in the field of transcriptomics (Kobak and Berens, 2019).

Importantly, our investigation will focus on the representation of brain networks generated in different studies with different data acquisition protocols, scanners, and populations. This is an initial step in evaluating the potential of these techniques to markedly improve visualization, potentially contribute to quality control, and ultimately lead to new interpretations of brain functional networks.

## Materials and Methods

### Participants

The current study used two different datasets to demonstrate the ability to combine data across studies using low-dimensional manifold methods. Data from the Human Connectome Project (Van Essen et al., 2013) (HCP) were used in the manifold learning step. These data are publically available and can be used without subject’s consent. A separate data set from a prior study examining aging and alcohol consumption performed in our laboratory (Moussa et al., 2015; Mayhugh et al., 2016) was used for an independent test embedding. These data were collected using procedures approved by the Institutional Review Board at Wake Forest School of Medicine. All participants gave written informed consent prior to participating in the research protocol. Both studies had fMRI data from resting-state and from a 2-back working memory task, though there were notable differences in the MRI sequences (described below) and the task designs.

### Data Examples for Working Memory vs. Resting State Connectivity Study

#### Example Data 1: Human Connectome Project S1200 Database

The HCP data released to date include 1,200 individuals. Of those, 1,113 (606 females; 283 minority) have complete MRI images, cognitive testing, and detailed demographic information (see Table 1). The current project used the minimally processed fMRI data provided by the HCP (Glasser et al., 2013) for resting state and working memory. The 830 subjects used are what remained after quality control assessment of head motion and global signal changes for both scan types. The HCP performed extensive testing and development to ensure comparable imaging across sites (Van Essen et al., 2012). The BOLD-weighted images were collected using the following parameters: TR = 720 ms, TE = 33.1 ms, voxel size 2 mm × 2 mm × 2 mm, 72 slices, 1,200 volumes.

**TABLE 1:** Basic demographic characteristics of both cohorts.

	HCP 830		Aging younger		Aging older		Aging combined	
Total subjects	830		22		41		63	
**Sex**								
Male	385 (46.4%)		10 (45.5%)		22 (53.7%)		32 (50.8%)	
Female	445 (53.6%)		12 (54.5%)		19 (46.3%)		31 (49.2%)	

	**Avg**	**Std**	**Avg**	**Std**	**Avg**	**Std**	**Avg**	**Stdv**

Age	28.7	3.7	27.3	3.3	70.8	3.6	55.6	21.0
Education	15.0	1.7	19.2	2.2	16.4	2.5	17.4	2.8
Working memory performance (%)	87.4	9.8	96.3	3.8	78.3	23.2	84.6	20.7

**Race**	**Total**		**Total**		**Total**		**Total**	

Am. Indian/Alaskan Nat.	2		0		0		0	
Asian/Nat. Hawaiian/Othr Pacific Is.	53		3		0		3	
Black or African Am.	97		1		2		3	
White	637		16		38		54	
More than one	23		0		1		1	
Unknown or not reported	18		2		0		2	

**Ethnicity**	**Total**		**Total**		**Total**		**Total**	

Hispanic/Latino	77		2		0		2	
Not Hispanic/Latino	742		20		41		61	
Unknown or not reported	11		0		0		0	

#### Example Data 2: Wake Forest School of Medicine Aging and Alcohol Consumption Database

Data in this study was collected as part of a prior study examining the effect of the interaction between age and alcohol consumption on brain networks (Moussa et al., 2015; Mayhugh et al., 2016) in community dwelling participants. The dataset is comprised of forty-one older adults [65–80 years old, sex (M/F) = 22/19] and twenty-two younger adults [24–35 years old, sex (M/F) = 10/12] who consumed alcohol across a range of consumption levels. All participants had brain imaging completed on a 3T Siemens Skyra scanner in a single visit. T1-weighted structural data were acquired in the sagittal plane using a single-shot 3D MPRAGE GRAPPA2 sequence (resolution = 0.98 × 0.98 × 1.0 mm, acquisition time: 5 min and 30 s, TR = 2.3s, TE = 2.99 ms, 192 slices). Resting-state as well as 1-back and 2-back working memory fMRI data (resolution = 3.75 × 3.75 × 5.0 mm) were acquired for each participant using BOLD-contrast images in an echo-planar imaging sequence (acquisition time = 6 min and 20 s, TR = 2.0s, TE = 25ms, flip angle = 75*^o^*, volumes = 187, slices per volume = 35). The resting-state and 2-back working memory scans are used in the current study to compare with the resting-sate and 2-back data from the HCP.

#### Description of the Tasks Performed During fMRI Data Acquisition

Participants in the HCP completed two resting-state scans and two working memory scans. The two scans were collected with different phase encoding (right to left vs. left to right). The resting-state scans were collected back-to-back while participants quietly viewed a fixation point. The 2-back task was a block design that interleaved the 2-back condition with a 0-back condition and a rest period. The working memory task utilized photos, and different blocks had different photo types (faces, body parts, houses, and tools). Participants were alerted prior to each block to indicate the task type. For the 2-back they were instructed to respond anytime the current stimulus being presented matched the stimulus two trials back. The aging study collected a single resting-state scan while participants quietly viewed a fixation cross. For the 2-back task, white letters were sequentially presented on a black background. Participants were asked to respond with either a right (yes) or left (no) finger press to indicate if the letter they were currently viewing was the same letter that was presented two letters before. The task was presented in continuous fashion with no alternating blocks.

### Structural and Functional MRI Processing

#### Human Connectome Project S1200 Database

The HCP data is currently available in multiple stages of processing. Data run through the Minimally Processed pipeline (Glasser et al., 2013) were used. In addition, the data were motion corrected using ICA-AROMA (Pruim et al., 2015), a method that automatically and robustly classifies the output of MELODIC, the first 14 volumes were removed from each scan, and band-pass filtering (0.009–0.08 Hz) was applied to remove physiological noise and low-frequency drift.

The block design of the working memory task required some additional processing before networks could be generated specific to the 2-back condition. The block design was modeled in SPM12 using the Specify 1st-Level tool, providing regressors for 0-back and rest blocks along with the cues for uses in the final regression analysis. The modeling of these elements of the block design allowed us to remove any persisting unwanted signals that bleed into the 2-back blocks. Considering each scan was collected twice with opposite phase encoding, the two scans were concatenated and accounted for with the inclusion of a scan-specific regressor. Additional regressors included the average gray matter (GM), white matter (WM), and cerebrospinal fluid (CSF) signals along with the realignment parameters All regressors were used in a single regression analysis and the residual signal aligned with the 2-back blocks were then extracted and concatenated into a single time series. To ensure that any differences observed between the rest and task scans were not due to the extraction and concatenation of the individual 2-back blocks, the exact same time points (from the beginning of the series) that were used for the 2-back scan were extracted from the resting state scans and concatenated. This process resulted in 268 functional volumes to be used for generated functional brain networks described below.

#### Wake Forest School of Medicine Aging and Alcohol Consumption Study

Standard image preprocessing was conducted using SPM12^1^. Structural images were segmented into six tissue probability maps: GM, WM, CSF, bone, soft tissue, and air/background. GM and WM maps were combined to create a brain tissue map. This image was warped using Advanced Normalization Tools (ANTs) (Avants et al., 2011) to Colin space^2^ to match the Shen atlas (Shen et al., 2013). The inverse transform produced by ANTs was applied to the atlas in order to put the atlas into the native space of each subject. Structural images were then co-registered to each functional image. Resulting transforms were applied to segmentation maps as well as the native space atlas. Other preprocessing of the functional data included: discarding the first 10 volumes to ensure that fMRI signals had achieved equilibrium, slice time correction, realignment to the first volume, band-pass filtering [0.009–0.08 Hz (Power et al., 2012; Yamashita et al., 2018)], and regressing six rigid-body transformation parameters that were generated during the alignment process along with average brain tissue signals (GM, WM, and CSF). Functional data were motion corrected using ICA-AROMA (Pruim et al., 2015). Because the 2-back task was collected in continuous fashion with no alternating blocks, the time-series clipping and concatenation procedures used for the HCP data were not necessary.

#### Functional Network Generation

Although acquisition and preprocessing differed between the HCP and Aging studies, network generation was the same for both datasets. After preprocessing, the brain was parcellated into 268 regions as defined in the Shen Atlas (Shen et al., 2013) by averaging the BOLD signal from all voxels within each region for each participant. A functional network was constructed for each participant by computing the Pearson (full) correlation between the resultant time series for each region pair. All subsequent analyses used fully connected weighted networks, comprising both positive and negative connections.

#### T-Stochastic Neighbor Embedding

T-stochastic neighbor embedding is a non-linear machine learning algorithm developed for visualization of high-dimensional data. It is an unsupervised algorithm that projects high-dimensional data into a lower space in two main steps. First, a probability distribution over high-dimensional pairs points is constructed such that similar (high-dimensional) points get higher probabilities. Then, a t-Student probability distribution over low-dimensional data is constructed, and the Kullback-Leibler divergence between the two distributions is minimized to obtain the final low-dimensional points locations after sufficient number of optimization iterations.

We used an extended version of t-SNE called Fit-SNE (Linderman et al., 2019) that is much faster than the original algorithm. We based our implementation on the examples provided on the software’s website^3^. The initialization was set to the PCA of the training data with 50 components, but the original data was passed to fast_tsne to be embedded. The learning rate was set to the number of subjects divided by 12. Two values were provided for perplexity as a list, 30 and the number of subjects divided by 100.

#### Uniform Manifold Approximation Projection

We also used UMAP, a recent breakthrough in the field of manifold learning, to embed the functional brain networks onto a low dimensional manifold. A detailed description of UMAP and the underlying theory have previously been presented (McInnes et al., 2018). Here, we will briefly describe general technical details of the algorithm and main parameters. The two main assumptions behind UMAP are: (1) the data is uniformly distributed and (2) there is local connectivity. While in practice data rarely behave uniformly, it is possible based on properties of topological spaces to find metrics and representations that approximately meet this assumption. Local connectivity implies that no point is isolated. Overall UMAP generates: (1) a manifold approximating the data in the high-dimensional space by creating and patching local fuzzy simplicial sets (Spivak, 2012) into a topological representation of the data; (2) a similar representation in the low dimensional manifold onto which the data is to be embedded and (3) an optimized layout of the data representation in the low dimensional space by minimizing the cross-entropy between the two topological representations.

Uniform manifold approximation projection has several essential hyper-parameters: (1) the dimension (d) of the low dimensional manifold where the data will be embedded; for visualization *d* = 2–3 but for dimension reduction larger values can be selected; (2) a metric or distance in the high-dimensional space (e.g., Euclidean and Minkowski, etc); (3) the number of neighbors (k) to use when constructing the topological approximations of the data; and (4) the minimum distance (min_dist) which is the desired separation between close points in the embedding space.

In our study we used the latest available version of UMAP (0.4) on a computer running Red Hat 7.6 with Python 3.7 installed. The parameters used in this work to create UMAP embeddings were as follows: number of neighbors = 15, min_dist = 0.0, *d* = 2, random state = 42, and transform seed = 42. We set the repulsion strength (gamma) to 0.45 for the transformation of new data to the UMAP defined space. Any parameters not listed were kept at their default values.

Extensions of UMAP and t-SNE have been developed to allow the extension of new data onto an already existing low dimensional embedding. For UMAP the new data point is positioned using a weighted average position of the k-nearest neighbors of the training data embedding (McInnes et al., 2018). The same optimization step previously used to embed the training data is applied to the new data point but keeping fixed the data points corresponding to the training embedding which optimizes the position of the new point with respect to them. To guarantee reproducibility of results, the random state in the call to UMAP function and the random seed in the transformation were set to the same values. For t-SNE we used a procedure described in Kobak and Berens (2019) which for each new data point the k-nearest neighbors among the training networks in the high-dimensional space are selected, using Pearson correlation as distance. Then in the 2D map a new data point is positioned at the median location of the corresponding k reference networks as embedded by t-SNE during training.

#### Mapping Brain Networks to Two Dimensional Space

**FIGURE 1:**
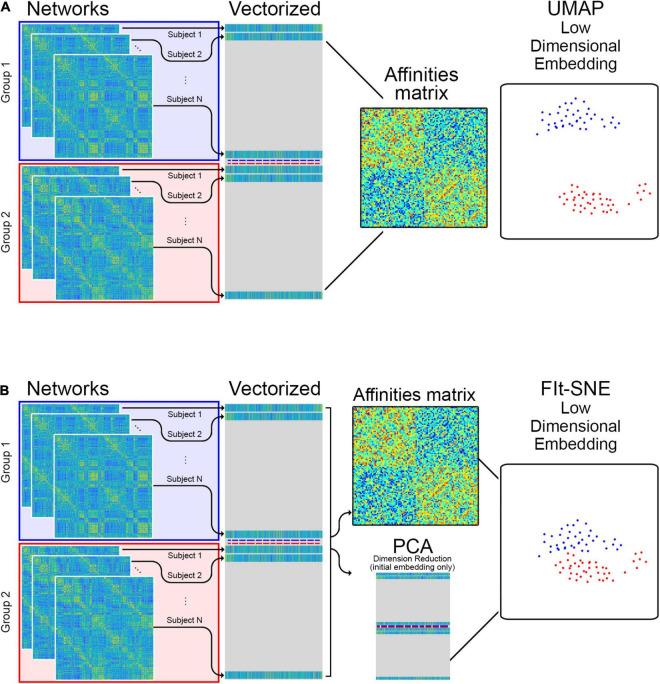
**(A)** The edge information of individual networks is vectorized and stacked in a matrix which is provided as input to UMAP for low dimensional representation. **(B)** For the Fit-SNE version of t-SNE, dimension reduction using principal components analysis (PCA) is applied to the vectorized, stacked matrix during the initial embedding. Subsequently, the embedding is performed directly on the vectorized, stacked matrix.

Figure 1 illustrates how the networks are provided as input to t-SNE and UMAP. The matrices containing the edge information (correlations) from all individuals in each group are vectorized and stacked in a matrix where each row corresponds to a network from a specific individual and each column correspond to edges between two brain regions across individuals. This matrix is directly input to UMAP algorithms and t-SNE. To investigate the performance of both approaches embedding brain networks into 2D space, we designed 4 different experiments based on data from both studies (HCP and aging study). We embedded resting state and memory task networks from: (1) 830 subjects available in the HCP project; (2) 63 subjects available in the aging study; (3) all subjects in both studies combined. Finally, in (4) we transformed the networks from the aging dataset into previously existing embeddings of the HCP networks. These transformations were accomplished using extensions that have been developed for UMAP and fit-SNE.

To characterize the performance of both algorithms we used two metrics previously used in transcriptomics data analysis (Kobak and Berens, 2019): (1) KNN - the fraction of k-nearest neighbors in the original high-dimensional data that are preserved as k-nearest neighbors in the embedding and (2) CPD - Spearman correlation between pairwise distances in the high-dimensional space and in the embedding. In addition, we used Random Forests for classification (Breiman, 2001) to quantify the discrimination of the representation of the two types of brain networks in the low dimensional space. We used the implementation available in the randomForestSRC R package (Ishwaran and Kogalur, 2014).

## Results and Discussion

**FIGURE 2:**
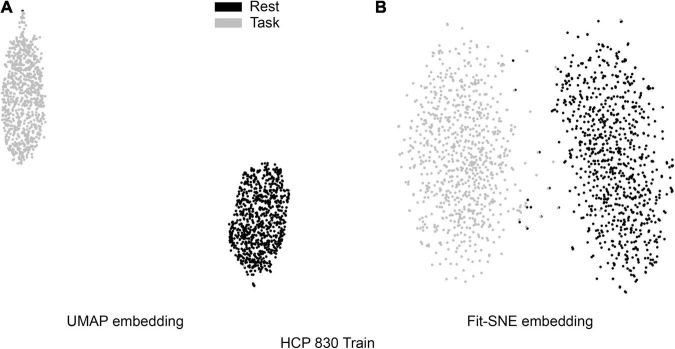
**(A)** UMAP’s embedding of the resting state and 2-back networks corresponding to 830 HCP participants is shown. **(B)** The corresponding representation generated by fit-SNE is presented.

**FIGURE 3:**
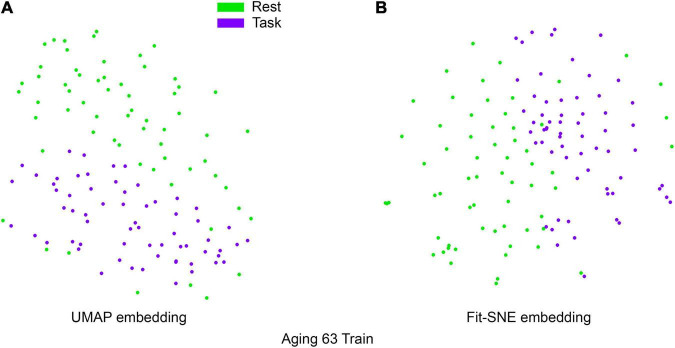
**(A)** UMAP’s embedding of the resting state and 2-back networks corresponding to 63 participants of the aging study is shown. **(B)** The corresponding representation generated by fit-SNE is presented.

**FIGURE 4:**
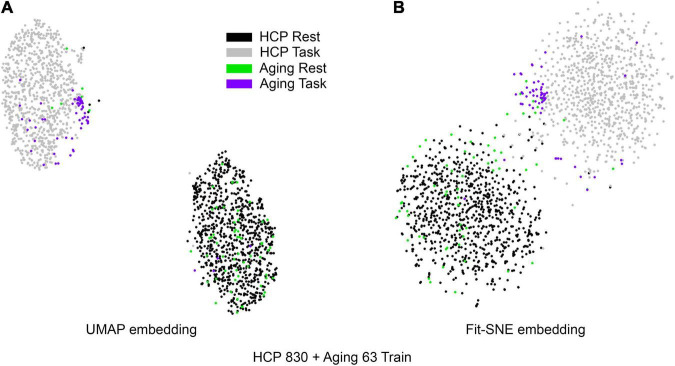
**(A)** UMAP’s embedding of the resting state and 2-back networks from both studies combined is shown. **(B)** The corresponding representation generated by fit-SNE is presented.

**FIGURE 5:**
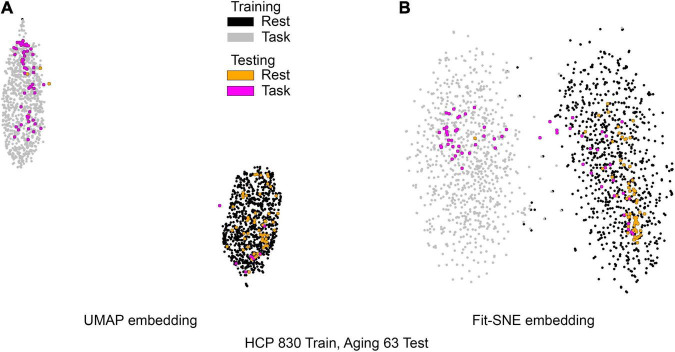
**(A)** UMAP’s embedding of the resting state and 2-back networks from both studies is shown. In this case the brain networks from the aging dataset were projected into the already existent embedding of HCP brain networks. **(B)** The corresponding representation generated by fit-SNE is presented.

Table 1 presents the basic demographic characteristics of both cohorts. The HCP participants overall were much younger and scored higher in the memory task test compared to the Aging study. The HCP cohort had a larger proportion of females and in both studies White participants were a majority. UMAP and t-SNE performance, in the four situations described above, is illustrated in Figures 2–5 where the low dimensional representation of the resting state and 2-back brain networks are presented. In Figure 2A, UMAP’s embedding of the resting state and 2-back networks corresponding to 830 HCP participants is shown. In panel B the corresponding representation generated by fit-SNE is presented. Both approaches generated low dimensional representations where the two different types of networks could be discriminated with high levels of accuracy (99.8 and 98.6%, respectively). Figure 3 presents similar results for the Aging study data. Although the accuracy of discrimination achieved by Random Forests is not as high as for the HCP dataset, it is still high (88.1 and 87.3%, respectively). It is possible that this is due to the much smaller sample size and more heterogeneous nature of the aging study dataset. Finally, Figures 4, 5 present the performance of UMAP and fit-SNE when embedding the combined datasets and when projecting the Aging study dataset onto the already existing embedding of the HCP dataset. In both cases the accuracy of discrimination was very high, indicating that the type of brain networks of both studies aligned most of the time correctly in the low dimensional manifold. Overall both approaches were able in all situations to identify two large clusters of brain networks derived from resting state and memory task data. The KNN and CPD metrics showed that t-SNE more often tended to preserve the neighbors and relative distances in the high-dimensional space after their extension onto the 2D space. It is important to only compare within rows the table between methods due to differences in dataset sizes that can influence these metrics (see Table 2). However, UMAP clearly generated low dimensional representations with higher discrimination of the types of brain networks (see Table 3) evaluated using a machine learning classifier. We confirmed here that the use of PCA initialization by t-SNE and spectral embedding by UMAP make both approaches less dependent of random seeds increasing the reproducibility of the results. We repeated the experiment corresponding to the first entry of Table 2 using 20 different random seeds observing very small variability of the results.

**TABLE 2:** Metrics evaluating the preservation of the networks topological Relationships are presented.

UMAP			T-SNE		
Dataset	KNN-ratio	CPD	Dataset	KNN-ratio	CPD
HCP 830	0.10	0.43	HCP 830	0.14	0.45
Aging	0.27	0.34	Aging	0.30	0.36
Combined	0.08	0.38	Combined	0.13	0.40
HCP < −Aging	0.25	0.14	HCP < −Aging	0.18	0.09

**TABLE 3:** Results of classification of the brain networks in the embedding space using RF are presented.

	UMAP			Fit-SNE		
Dataset	Acc (%)	Sens. (%)	Spec. (%)	Acc (%)	Sens. (%)	Spec. (%)
HCP 830	99.8	99.8	99.9	98.6	98.4	98.7
Aging	88.1	88.9	87.5	87.3	93.7	81.0
HCP830 + Aging	98.9	98.8	99.1	97.7	98.2	97.2
HCP830 < −Aging	99.9	98.9	99.1	97.3	96.1	98.4

In this work we have used a recent breakthrough in manifold learning, UMAP, to represent functional brain networks generated by two different studies independently and combined in a common low dimensional space. We also tested t-SNE, a manifold learning technique, considered to be for several years the state the art in the field. While t-SNE has been used more often to visualize and investigate neuroimaging data including fMRI, UMAP’s capabilities to deal with fMRI high-dimensional data are less known. To the best of our knowledge this work is one of the first instances where UMAP’s potential to represent and visualize functional brain networks in a low dimensional space has been tested.

Our work demonstrates the feasibility of projecting networks generated by different studies in a common low dimensional space. We have used a well-known and publicly available to the neuroimaging community dataset, the Human Connectome Project and a study developed in our lab (both independently and combined), to derive and project functional brain networks into a two dimensional space. This resulted in an excellent discrimination of resting state networks from 2-back networks. This high level of performance for co-embedding data across different studies was achieved despite dramatic differences in the details of the 2-back task, differences in the MRI scanners and imaging protocols, and differences in study-specific image preprocessing steps.

This opens new possibilities for functional brain networks visualization, dimension reduction, and possible meta-analyses across studies. The possibility of a quick and simple visualization of such complex datasets as fMRI brain networks in 2 or 3 dimensions has the potential to allow identification of structure or particular features like outliers within the data. Quality control is an another area of potential application. We observed that networks corresponding to some individuals fell into the wrong cluster. While here we were not able to determine abnormalities in these specific datasets collected in the past, this could be useful for networks generated in the future. A tool like this could be part of the quality control process. New datasets can be run through it, and falling into the wrong cluster could be a red flag signaling possible data quality problems. These could be checked via the repetition of the scan or through thorough examination of the fMRI data and its processing. Other possibilities could be the discovery of specific individuals whose networks are really different from the population being compared or the detection of wrong settings of the transformations parameters. These are topics that are beyond the scope of this project but deserve more research.

There seem to be polemical views in the field of transcriptomics about which method (UMAP or t-SNE) is better (Becht et al., 2018; Kobak and Berens, 2019). While McInnes et al. (2018) have claimed the superiority of UMAP preserving global structure existent in the ambient space, Kobak and Berens (2019) have presented computational experiments suggesting that when using a proper initialization in the low dimensional manifold both approaches perform similarly preserving global structure. Overall this comparison is complex and very difficult to do fairly since both approaches have multiple parameters than can be tuned, and perhaps this also could depend on the nature of the data in each specific problem. It was not our goal to perform an exhaustive comparison of the two approaches but rather to explore the feasibility of transforming brain networks data from different studies into a common 2D space. Our results clearly suggest that UMAP, a more recent technique, is an effective data reduction method for neuroimaging studies. However, our 2D analyses did not show it to be superior to t-SNE. All our statements about performance of both approaches are based on the specific settings of parameters we selected and do not represent a rigorous comparison. It is important to note that UMAP is designed to be a dimension reduction technique to any dimension while t-SNE usually is used in 2D-3D settings.

An important takeaway from our work is the effectiveness of methods based on data topology to deal with high- dimensional data and specifically in this case functional brain networks (> 30K variables or edges). Previously some groups have used data topology principles to analyze fMRI data such as the Mapper algorithm and persistent homology (Carlsson, 2009; Saggar et al., 2018; Chung et al., 2019; Geniesse et al., 2019). These are topological data analysis tools used for analyzing point cloud data that show great promise. Since these techniques are relatively new to the neuroimaging community, we expect this study and our experiences reported here will be helpful to brain imaging researchers interested in data reduction and high-dimensional data visualization and analysis.

This work is not without limitations. We did not perform a more exhaustive comparison of the impact of hyperparameters setting on the 2D representations. Our analyses were based on the metrics provided by the software packages. Our experiments were limited to two types of networks resting state and a memory task that were present in both of the studies that we used. While this limits the generalizability of this work, it is our anticipation that this method will be used more widely to compare a wide range of tasks. Also it should be kept in mind that in general fully representing the complexity of high-dimensional spaces in two or three dimensions is not possible. For example, in a 15 dimensional space there could be found 16 equidistant points that are not possible to translate to 2D or 3D spaces (Van Maaten and Histon, 2008). Also the curse of dimensionality first reported by Richard Bellman (Bellman, 1961) is associated with non-intuitive properties of high-dimensional spaces (Hastie et al., 2001; Cherkassky and Mulier, 2007) which constitute a challenge when translating high-dimensional data to 2D or 3D spaces. More research is needed to determine the utility of the data generated by UMAP and fit-SNE. The metrics or distances that are used by UMAP and fit-SNE are suboptimal and do not take into account the networks structure and topology. Finding metrics based Riemannian or topological distances (Chung et al., 2017; Venkatesh et al., 2020) between networks or is a promising area for future research

Further research is also needed to apply these methods to dynamic brain networks due to differential within and between subject variability. Finally, there is a clear difference between manifold learning techniques (that can reduce data to an arbitrary dimension) and those specifically intended for visualization in 2D or 3D such as t-SNE (and UMAP most popular usage). Visualization is a useful tool that can uncover complex high-dimensional structure and can accelerate data exploration, bringing benefits to the neuroimaging community to make new scientific discoveries. However, these tools should be used with caution. Information is usually lost when transforming the data into 2D which could lead to misleading analyses or conclusions especially if the hyperparameters are not properly tuned.

## Conclusion

We have investigated the performance of two high-dimensional data visualization techniques (t-SNE and UMAP) considered to be the state of the art in the field of manifold learning when transforming brain functional networks into 2D spaces. We have found that they are able to efficiently detect structure in the network data derived from fMRI collected in the resting-state and during working memory tasks. This was possible even when the data from two studies were combined, despite dramatic differences in nearly every aspect of the data including but not limited to the MRI scanners, imaging and task protocols, study populations/demographic differences, and study-specific image preprocessing steps. Finally, we demonstrated that learning the manifold with one dataset allowed the embedding of a novel dataset without requiring modification of the learned parameters. This may be useful for meta-analyses and for future work that uses embedding of individual brain networks for clinical applications such as diagnoses or classification of specific brain conditions.

## Data Availability Statement

Publicly available datasets were analyzed in this study. The human connectome data can be found here: https://www.humanconnectome.org/study/hcp-young-adult. The aging data used can be found here: http://fcon_1000.projects.nitrc.org/indi/retro/wakeforest_lcbn_alcohol_aging.html.

## Author Contributions

RC, RL, and PL conceived and designed this study and drafted the manuscript. RL and RC performed the analyses. RL, MB, and SS completed preprocessing and quality control for the HCP data. All authors participated in the interpretation of the results, revised the draft critically for important intellectual content, gave final approval to the submitted manuscript, and agreed to be accountable for all aspects of the work.

## Conflict of Interest

The authors declare that the research was conducted in the absence of any commercial or financial relationships that could be construed as a potential conflict of interest.

## Publisher’s Note

All claims expressed in this article are solely those of the authors and do not necessarily represent those of their affiliated organizations, or those of the publisher, the editors and the reviewers. Any product that may be evaluated in this article, or claim that may be made by its manufacturer, is not guaranteed or endorsed by the publisher.

## Footnotes

1
www.fil.ion.ucl.ac.uk/spm/

2
http://www.bic.mni.mcgill.ca/ServicesAtlases/Colin27

3
https://github.com/KlugerLab/FIt-SNE/
